# Viral metagenomics revealed novel betatorquevirus species in pediatric inpatients with encephalitis/meningoencephalitis from Ghana

**DOI:** 10.1038/s41598-019-38975-z

**Published:** 2019-02-20

**Authors:** Daniel Eibach, Benedikt Hogan, Nimako Sarpong, Doris Winter, Nicole S. Struck, Yaw Adu-Sarkodie, Ellis Owusu-Dabo, Jonas Schmidt-Chanasit, Jürgen May, Daniel Cadar

**Affiliations:** 1 Department of Infectious Disease Epidemiology, Bernhard Nocht Institute for Tropical Medicine, Hamburg, 20359 Germany; 2 German Center for Infection Research, Hamburg-Borstel-Lübeck-Riems, Borstel, 20359 Germany; 3 Kumasi Centre for Collaborative Research in Tropical Medicine, Kumasi, 40080 Ghana; 4 Department of Clinical Microbiology, Kwame Nkrumah University of Science and Technology, Kumasi, 40080 Ghana; 5 Department of Arbovirology, Bernhard Nocht Institute for Tropical Medicine, Hamburg, 20359 Germany

## Abstract

The cause of acute encephalitis/meningoencephalitis in pediatric patients remains often unexplained despite extensive investigations for large panel of pathogens. To explore a possible viral implication, we investigated the virome of cerebrospinal fluid specimens of 70 febrile pediatric inpatients with clinical compatible encephalitis/meningoencephalitis. Using viral metagenomics, we detected and genetically characterized three novel human Torque teno mini virus (TTMV) species (TTMV-G1-3). Phylogenetically, TTMV-G1-3 clustered in three novel monophyletic lineages within genus *Betatorquevirus* of the *Anelloviridae* family. TTMV-G1-3 were highly prevalent in diseased children, but absent in the healthy cohort which may indicate an association of TTMV species with febrile illness. With 2/3 detected malaria co-infection, it remains unclear if these novel anellovirus species are causative agents or increase disease severity by interaction with malaria parasites. The presence of the viruses 28 days after initiating antimalarial and/or antibiotic treatment suggests a still active viral infection likely as effect of parasitic and/or bacterial co-infection that may have initiated a modulated immune system environment for viral replication or a defective virus clearance. This study increases the current knowledge on the genetic diversity of TTMV and strengthens that human anelloviruses can be considered as biomarkers for strong perturbations of the immune system in certain pathological conditions.

## Introduction

In sub-Saharan Africa, fever remains one of the most common symptoms of acute pediatric illness and is the main reason for seeking health-care advice. Acute febrile illness is responsible for majority of childhood deaths after the neonatal period and under-5 years^1,2^. Acute central nervous system (CNS) infections such as encephalitis/meningoencephalitis are complex, sometimes fatal neurologic conditions accompanied by high-grade fever and characterized by inflammatory lesions in the brain with high morbidity and mortality in children, which are especially prone^3^. One of the most common causes of pediatric impaired consciousness in sub-Saharan Africa is cerebral malaria^4^. Although up to 70% of pediatric patients in sub-Saharan Africa^5^ exhibit asymptomatic malaria parasitaemia, other possible causes of encephalitis/meningoencephalitis (e.g., viral or bacterial CNS infection) must be excluded in order to confirm cerebral malaria^4^. Thus, a complex analysis of the viral composition in acute CNS infection cases is crucial to better understand the possible causes or interactions within these pathological conditions to improve the clinical management of the patients and ultimately reduce disability-adjusted-life-years (DALYs) due to implementation of prevention and treatment programs. Advances in next generation sequencing (NGS) technology have revolutionized the virus discovery and the possibility to deeply explore the virome in clinical specimens such as sera, whole blood, cerebrospinal fluid, respiratory swabs and diarrheal stools. Recent studies have shown that NGS is a powerful tool for identification of viral pathogens in encephalitis/encephalopathy patients by detecting DNA/RNA viruses in cerebrospinal fluid (CSF) or brain tissue^6,7^. The goal of the present study was to use viral metagenomics in order to explore the possible viral implication in CNS infections by determining the cerebrospinal fluid virome profile of 70 acute febrile pediatric inpatients with compatible encephalitis or meningoencephalitis from rural Ghana consulting medical advice.

## Results

### Identification of viral sequences in clinical samples using NGS

**Figure 1:**
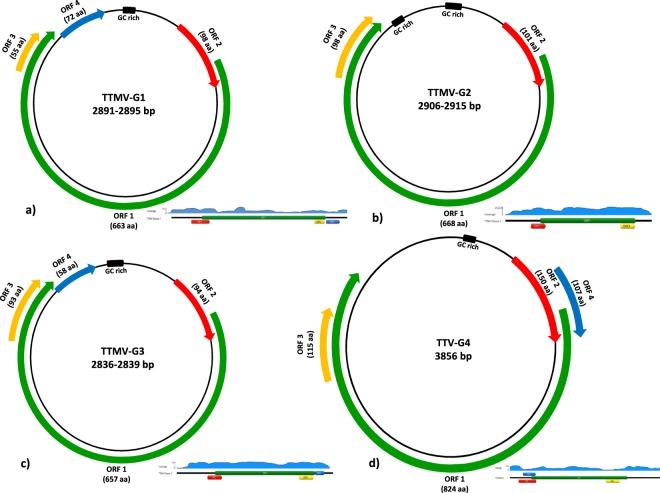
Genomic organization of the TTMV-G1–3 (**a**–**c**) and TTV-G4 (**d**) from pediatric patients with CNS infection. The circumference of circles of each virus species represents the relative size of the genome. The arrows represent the relative sizes, directions and reading frame of each putative ORF (ORF1-ORF4). A closed black box indicates the GC-rich region. Coverage of the NGS raw reads for each anellovirus species is indicated. For better visualization of the coverage the circular genome is linearized. The coverage plots are informative and represent each one individual patient.

A total of 70 febrile severely ill pediatric inpatients with clinical signs compatible for encephalitis/meningitis where cerebrospinal fluid (CSF) was collected, were enrolled for viral metagenomics analysis. CSF samples were first pooled as 3 samples/pool and included in the library preparation pipeline. The libraries obtained were sequenced using MiSeq sequencing platform. The raw reads were then assembled into contigs and with the unassembled singlets were compared with viral proteome and domain database using BLASTx and an E-value < 0.001. Viral sequences related to several anelloviruses have been detected in CSF samples. The anellovirus-like contigs were assembled and 4 complete anellovirus genomes were successfully recovered (Fig. 1). No additional reads related to other viruses were observed in the analyzed samples. The library obtained from the negative water control do not contained neither anellovirus nor any viral reads. In addition, the CSF and sera samples subjected to NGS have been previously detected negative by using a large panel of viral RT-PCRs tests but 2/3 was positive for malaria^8^. In our metagenomic sequencing we were also able to recover the previously detected three pathogens (*S. pneumoniae*, *H. influenzae*, *P. falciparum*)^8^.

### Genomic organization and sequence analysis of TTMV-G1-3 and TTV-G4

**Figure 2:**
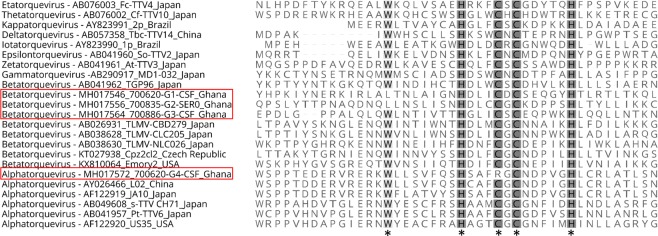
Amino acid alignment of the ORF2 showing the conserved amino acids within the common motif WX_7_HX_3_CXCX_5_H (shaded and marked with star) among the TTMV-G1–3, TTVG4 and representative members of *Betatorquevirus*, *Alphatorquevirus* and other genera of the *Anelloviridae* family.

Complete genome sequences of the newly discovered anelloviruses from all positive patients’ CSF specimens were successfully obtained using NGS, inverse PCR with specific primers and Sanger sequencing using primer walking (primers sequences are available upon request). The complete genome of TTMV-G1 was 2891–2895 bp in length and contained four open reading frames (ORFs) (Fig. 1). Similar genomic organization with a genome size of 2836–2839 bp was observed for TTMV-G3, while TTMV-G2 exhibited a genome with three ORFs and a genome size of 2906–2915 bp (Fig. 1a–c). The variable genome sizes were due to the source of samples being from different patients. No intrapatient variability of genome size has been observed. The genome of TTV-G4 was 3856 bp in length and consisted of four major ORFs (Fig. 1d). The UTR region of the genome was the most conserved region including the characteristic GC rich region (Fig. 1). The genomic organization, nucleotide and amino acid length of the putative ORFs are shown in Fig. 1. These characteristics resemble the genomic features of human Torque Teno mini viruses and Torque Teno viruses. The transcribed elements (TATA-box, Sp1 site, Cap site, poly(A) signal) were conserved in all TTMV-G1-3 and TTV-G4 genomes (Table 1). The sequence GGGGCAATT resembling the presumed cap site in the 5′-ends of mRNAs of human respiratory syncytial virus genes^9^ was conserved in all anelloviruses analyzed (Table 1). Interestingly, two conserved sequences of 15 nt (CGAATGGCTGAGTTT and AGGGGCAATTCGGGC) in the UTR previously reported in all TTMV and TTV were not maintained in all anellovirus genomes from the study. The torque teno/chicken anemia virus (CAV)-common protein-tyrosine phosphatase (PTP) motif WX_7_HX_3_CXCX_5_H^10–12^ was found in all ORF2 of TTMV-G1-3 and TTV-G4 described here with one position exchanged in TTMV-G1-2 (**L**X_7_HX_3_CXCX_5_H) and one in TTV-G4 (WX_7_HX_3_**R**XCX_5_H) (Fig. 2). A similar mutation has been recently observed in gorilla TTMV species^13^.

**Table 1:** Genomic characteristics, predicted open reading frames and locations of transcription elements of the torque teno virus species discovered in the hospitalized pediatric patients with encephalitis/meningoencephalitis.

TT Species	Genomic length (nt)	Coding region	Putative ORFs	TATA box (ATATAA)	Sp1 site (GGGCGG)	Cap site (GGGGCAATT)	Poly(A) signal (AATAAA)	GC-rich region
TTMV-G1	2889/2895	337/339–2725/2731	ORF1–4	150/153–155/158	231/236–238/241	268/274–276/282	2721/2727–2726/2732	2838/2844–2889/2895
TTMV-G2	2906/2915	337/343–2537/2543	ORF1–3	151/154–156/159	231/237–236/242	269/275–277/283	2739/2742–2741/2747	2804/2807–2906/2915
TTMV-G3	2836/2839	352/355–2669/2672	ORF1–4	118/119–123/124	245/248–250/253	283/286–291/294	2664/2667–2669/2672	2728/2731–2836/2839
TTV-G4	3856	227–3027	ORF1–4	52–58	139–144	177–185	3023–3028	3094–3233

The nucleotide positions are variable due to different genomic lengths within the same virus species.

### Phylogenetic relationships of the newly discovered anelloviruses

**Figure 3:**
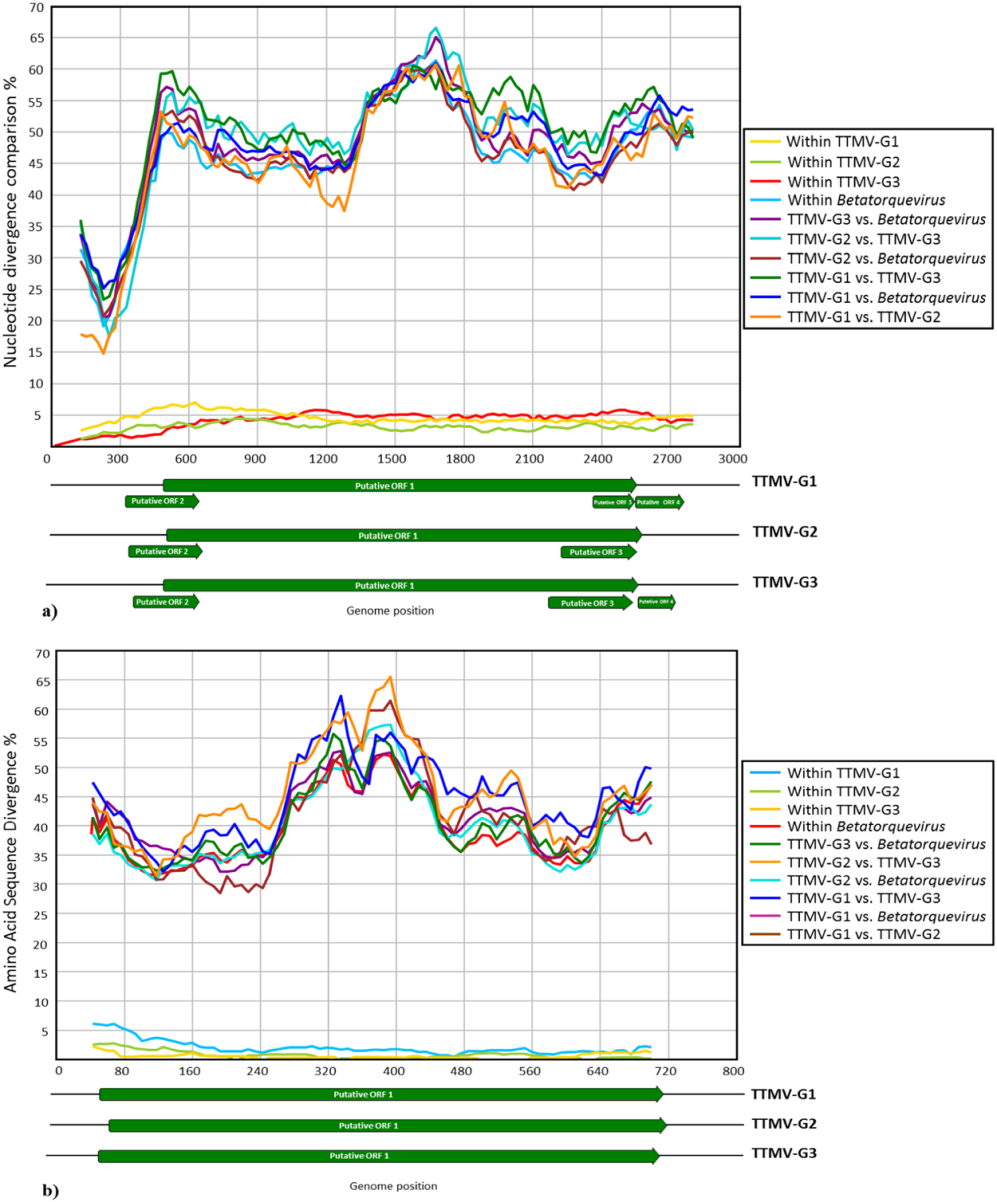
Nucleotide (**a**) and amino acid (**b**) sequence divergence scan of TTMVG1-G3 (uncorrected nucleotide and amino acid *p* distances) and representative members of *Betatorquervirus* genus using 250-nt fragments by 25-nt increments across the sequence alignment. Sequences were assigned into ten different tag groups. Within-TTMV-G1-G3, and within-*Betatorquervirus* distances are included for comparison.

**Figure 4:**
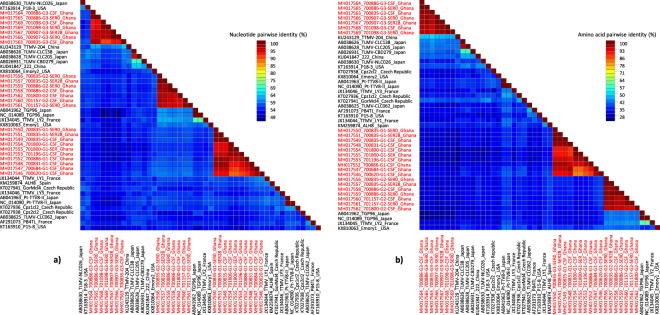
Colour-coded pairwise identity matrix generated from complete ORF1 nucleotide and amino acid sequences of the TTV-G1–3 and representative members of the *Betatorquervirus* genus. Each colored cell represents the percentage identity score between two sequences. The TTV-G1–3 strains are bolded in red. A color key indicates the correspondence between pairwise identities and the colors displayed in the matrix.

**Figure 5:**
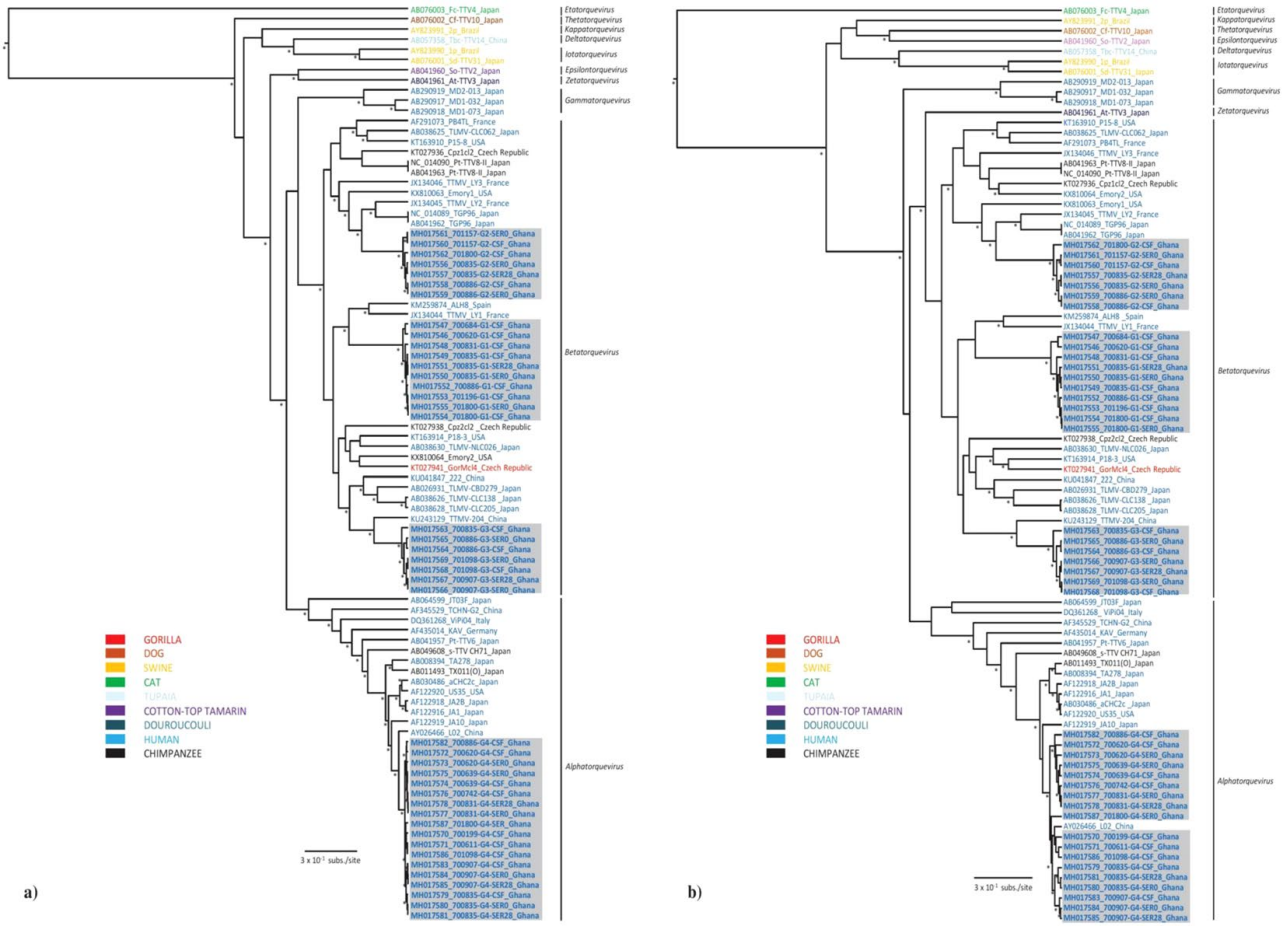
Bayesian maximum clade credibility (MCC) trees based on complete nucleotide (**a**) and amino acid (**b**) sequences of ORF1 showing the phylogenetic placement of the TTMV-G1-G3 and TTVG4 species from this study compared with representative members of the *Betatorquevirus*, *Alphatorquevirus* and other genera of the *Anelloviridae* family. Bayesian posterior probabilities (≥90%) and parallel maximum likelihood replicate percentages (≥70%) in which the associated taxa clustered in the bootstrap test (1,000 replicates) are indicated at the nodes (asterisk). The main lineages/genera are indicated to the right of the tree. Boldface indicates TTMV and TTV species from febrile pediatric patients (this study). Taxon information includes strain names, GenBank accession numbers, and countries of origin. The TTV strains are colored according to the host species. Scale bar indicates mean number of nucleotide/amino acid substitutions per site.

Due to the high divergence of the anelloviruses, the analysis of the entire ORF1 at the nucleotide level has been established as the most convenient approach for classification^14^. Thus, the nucleotide pairwise comparison facilitates identification of the genera and species levels. Based on the last report of the International Committee on Taxonomy of Viruses (ICTV)^14^, the classification of *Anelloviridae* is proposed with the following cut-off values for sequence divergence: genera >56%, species >35%. Our nucleotide pairwise identity comparison revealed that TTMV-G1-3 exhibit a nucleotide divergence between 39–49% (Figs 3 and 4). Given their sequence divergence, each can be assigned as a new human anellovirus species within the genus *Betatorquevirus* (Figs 3 and 4). The intraspecies nucleotide and amino acid divergence of the TTMV-G1-3 did not exceed 5% (Fig. 5). In addition, the complete genome of TTMVs obtained from serum and CSF samples from the same patient exhibited several non-synonymous mutations (0.3–0.9% divergence), which suggest that several variants of the same virus are present in the same patient. With a nucleotide identity of 91% to a previously described TTV in China, TTV-G4 is considered a new TTV variant (Fig. 5). To investigate the evolutionary relationships of TTMV-G1-3 and TTV-G4, a Bayesian phylogenetic analysis and maximum likelihood method was inferred based on nucleotide and deduced amino acid sequences of the putative ORF1 proteins including representative members of *Anelloviridae* genera (Fig. 5 ). The phylogenetic analyses revealed that TTMV-G1-3 belong to genus *Betatorquevirus* and does not cluster with any of the known TTMVs forming three highly supported distinct monophyletic lineages. All three novel TTMV species share common ancestor with relatives from Asia and Europe (Fig. 5). ORF1 nucleotide and protein distance matrix analysis confirmed the clear demarcation between TTMV-G1-3 and other members of genus *Betatorquevirus* (Fig. 4). Given their strongly supported distinct lineage clustering, we propose their inclusion as new TTMV species within the genus *Betatorquevirus* phylogeny (Fig. 5). TTV-G4 clustered within genus *Alphatorquevirus* phylogeny, and forms together with a Chinese relative a distinct monophyletic lineage (Fig. 5). Intra-host nucleotide divergence of the TTV-G4 ranged between 0.3–1%. Pairwise distance comparisons of TTMV-G1-3 and TTV-G4 nucleotide and amino acid sequences across the ORF1 are shown in Figs 3 and 4.

### Anelloviruses prevalence in febrile pediatric patients

The median age of the 70 recruited children was 28 months (IQR = 19–39) and sex was equally distributed, with 37 being female (53%). Based on the complete genome sequences, TTMV and TTV species specific primers (TTMV-G1-F: 5′-GGAGGGTGGAGTTGTAAAGT-3′, TTMV-G1-R: 5′-TGTGCTTTCTGAGTCTGTGG-3′; TTMV-G2-F: 5′-CTGCAAAGAGACTGGGACAT-3′, TTMV-G2-R: 5′-ATTCTTGTGGGGTAAAGCGG-3′; TTMV-G3-F: 5′-AACAACCCTGGAAACACCTC-3′, TTMV-G3-R: 5′-ACGTCTTCTTACCGTGTGTC-3′; TTV-G4-F: 5′-GGAGAAGGGGCAAAAAAAAG-3′, TTV-G4-R: 5′-GGGTGAATTTGTCTGTAGTC-3′) were designed to screen the CSF and serum samples from ill pediatric inpatients and serum specimens from healthy controls in order to investigate the possible association between the incidence of these viruses and encephalitis/meningoencephalitis cases. In total 14 out of 70 patients were positive for any anellovirus (Table 2). None of PCR positive febrile pediatric patients were tested negative to the sequencing using the TTMV specific primers. The TTMV-G1 virus species was detected in 10% (7/70), TTMV-G2 and TTMV-G3 in 6% (4/70) of both CSF and serum samples from pediatric inpatients, but absent in sera of the healthy control group (Table 2; Suppl. Table 1). TTV-G4 was detected in both, febrile ill children (16%) and afebrile healthy controls (3%) (Table 2; Suppl. Table 2). Furthermore, different combination of TTMV-G1-3 and TTV-G4 species co-infection were observed both in CSF and serum. In 2 meningoencephalitis cases associated with *S. pneumoniae* co-infection, the presence of all 4 anelloviruses was observed (Table 2). Except 2 febrile patients, all TTMV and TTV positive patients have been diagnosed with malaria or bacterial co-infection. The presence of anelloviruses in serum 28 days after hospitalization and initial antimalarial and/or antibiotics treatment was also being observed (Table 2). All positive CSF and serum samples from encephalitis/meningoencephalitis cases have further been subjected to complete genome sequencing by NGS and/or Sanger sequencing.

**Table 2:** The presence of TTMV-G1–3, TTVG4 and parasitic or bacterial co-infections in cerebrospinal fluid and serum of the hospitalized febrile pediatric patients.

Patient ID	Age (in months)	Gender	TTMV-G1	TTMV-G2	TTMV-G3	TTV-G4	*P. falciparum* count/µl	Blood culture isolate	CSF culture isolate
700611	24	female	—	—	—	CSF	130380	—	—
700620	36	male	CSF	—	—	SER0, CSF	—	—	—
700639	13	female	—	—	—	SER0, CSF	61740	—	—
700684	39	male	CSF	—	—	—	81224	—	—
700742	2	male	—	—	—	CSF	—	—	—
700831	55	female	CSF	—	—	SER0, SER28	241604	—	—
700835	159	male	SER0, SER28, CSF	SER0, SER28	CSF	SER0, SER28, CSF	—	—	*S. pneumoniae*
700886	2	female	CSF	SER0, CSF	SER0, CSF	CSF	—	*S. pneumoniae*	—
700907	20	female	—	—	SER0, SER28	SER0, SER28, CSF	26405	—	—
701098	30	male	—	—	SER0, CSF	SER0	139832	—	—
701157	38	female	—	SER0, CSF	—	—	351720	—	—
701196	144	female	CSF	—	—	—	—	*S. pneumoniae*	*S. pneumoniae*
701800	66	male	SER0, CSF	CSF	—	SER0	—	*H. influenzae*	*H. influenzae*
700199	11	male	—	—	—	CSF	130380	—	—

CSF - cerebrospinal fluid was collected at day 0 which corresponds to the first day of hospitalization; SER0 and SER28 are serum samples collected at first day of hospitalization and 28 days after initiating antimalarial and/or antibiotics treatment*. P. falciparum - Plasmodium falciparum; S. pneumoniae - Streptococcus pneumoniae; H. influenzae - Haemophilus influenzae*.

## Discussion

In sub-Saharan Africa (SSA), acute febrile illnesses associated with CNS infection remains one of the most common causes of childhood deaths after the neonatal period and under-5 years^1^. Nowadays, due to an implementation of effective prevention programs and efficient treatment of malaria infection in the early stage, the malaria-associated febrile illnesses and childhood mortality has dropped^15,16^. Recent studies showed that several non-malarial fevers (NMF) which represent an important cause of morbidity and mortality in pediatric patients in SSA can be attributed to viral and bacterial infections^17–21^. Nevertheless, the composition of the virome in febrile pediatric patients and its association with CNS infection is still poorly understood. In this study, we sought to elucidate the virome composition of the cerebrospinal fluid in children with high fever and clinical signs of CNS infection in a malaria-holoendemic area of the Asante Akim North District, Ghana. One third of the febrile pediatric patients enrolled in the present study were found malaria negative, thus the cause of their febrile condition could be attributed to other causes than malaria. Using viral metagenomics we detected 4 novel human anellovirus species in the CSF and serum from pediatric inpatients presenting with clinical signs of encephalitis or meningoencephalitis, mostly co-infected with malaria parasites or bacteria. However, the detection rate of the anelloviruses in comparison with the incidence of malaria was too low to provide any correlation between anellovirus co-infection and malaria. The first human torque teno virus (TTV) was discovered in the serum of a patient with post-transfusion hepatitis of unknown etiology in 1997 and was characterized as a small non-enveloped virus with a circular, single-stranded DNA of 3.8–3.9 kb^22^. Its discovery was followed by the detection of a large and diverse anellovirus population which has been classified in three genera, *Alphatorquevirus* including the Torque Teno virus *(TTV)*, *Betatorquevirus* with Torque Teno mini virus (TTMV) and *Gammatorquevirus* with torque teno midi virus (TTMDV) in the *Anelloviridae* family^14^. The genomes of anelloviruses vary between 2.1 to 3.9 kb in length, encode three or four overlapping ORFs and contain a characteristic GC-rich region in the UTR^23^. Although these viruses exhibit high level of genomic heterogeneity, as well apparent pan-tropism at the host level, the immunological properties and association of TTV in the etiology of specific diseases is unclear^24,25^. Chronic infection and co-infection with several virus species or genotypes are considered common features of anelloviruses^23^. Several studies suggest the implication of TTVs in hepatitis, different types of cancer, periodontitis, acute respiratory diseases and immune system disorders^22,26–29^. However, robust evidence about their disease-causing potential in the human populations, mostly due to their ubiquity is still missing. Phylogenetic analyses of the nucleotide and amino acid sequences of putative ORF1 show that TTMV-G1-3 belong to genus *Betatorquevirus* and does not cluster with any of the known TTMVs forming three highly supported distinct monophyletic lineages, while TTV-G4 clusters within genus *Alphatorquevirus*, and together with a Chinese TTV strain forms a distinct monophyletic lineage (Fig. 2). Given their strong lineage clustering and high nucleotide divergence, we propose the inclusion of TTMV-G1-3 as new TTMV species. A recent study suggests that intra-genomic recombination might be a cause of the heterogeneity of the TTV genomes^30^. However, in our study we were not able to detect any intra- or interspecies recombination events which might have led to the intra-host genetic diversity of the anelloviruses. The anelloviruses ORF2 protein has a conserved amino acid motif, WX_7_HX_3_CXCX_5_H which is located in the N-terminal that is also found in the CAV^31,32^. This conserved motif corresponds to the protein-tyrosine phosphatase (PTPase) signature motif, and has been suggested to be involved in the regulation of cellular and/or viral proteins during infection^33^. To determine whether the mutations observed in the motif of TTMV-G1-G2 and TTV-G4 from this study may confer an evolutionary advantage for the virus further studies are required. Although human anelloviruses are characterized by their high prevalence, with relatively uniform distribution worldwide in the healthy human populations, we were not able to detect any TTMV species among healthy children, with the exception of TTV-G4. The presence of these novel anelloviruses in serum 28 days after initial antimalarial and/or antibiotics treatment suggests a chronic infection, which is characteristic for TTVs^34^. The presence in serum of anellovirus mixed populations containing more than one species in one individual has been described^25^ and is considered a frequent event. In our study, the anellovirus co-infection seems to be a common feature in febrile children, where even in 2 patients all 4 anellovirus species were being observed. Furthermore, different combinations of viral species in different organs, suggestive of preferential tropism of certain TTV strains for specific tissues, have been reported^35,36^. Similar findings were observed in the present study, where different combinations of *Alphatorquevirus* and *Betatorquevirus* species co-infection were found in CSF and serum specimens. With only two out of 14 children having no other cause explaining the encephalitis symptoms, it remains uncertain if the TTVs contributed to the current febrile illness. However, it has to be kept in mind that the detection of *Plasmodium* parasites among children in a malaria endemic region does also not necessarily explain a febrile episode, as asymptomatic parasitaemia among healthy individuals is very common due to acquired immunity^37^. Interestingly, the new human TTMVs were only detected in febrile encephalitis children, but absent in the healthy cohort, which may indicate that the human TTMV species from this study might be associated with febrile illness in children. In contrast, TTV-G4 was common in febrile and afebrile children, thus a disease association is rather unlikely. The underlying pathomechanism by which these TTMV may cause disease remains unclear. In the present study, parasitic and/or bacterial co-infection may have created a permissive environment (immune system modulation) for anellovirus replication or a diminished virus clearance^38^. This is in line with previous observations which indicate a burden of anelloviruses in immunocompromised patients^39,40^. Hence, it has been suggested that these viruses could serve as biomarker to indicate the state of immunosuppression of an individual patient^39^. A previous study showed that the prevalence of anelloviruses in pediatric patient’s plasma increased with age until patients were 19–24 months of age followed by a decrease in prevalence^38^. These observations were most spectacular for TTV and TTMDV and very modest for TTMV^38^. Contrary to this study we did not observed any correlation between the prevalence and patient age. This might be due to geographic differences and diverse immune status of the studied pediatric patients. In general, the relative high proportion of acute encephalitis without a laboratory-confirmed etiology require next generation diagnostic techniques, such as metagenomics techniques, in order to better understand the complex etiology of these highly severe conditions. In conclusion, this study describes three novel TTMV species associated with CNS infection in febrile pediatric inpatients from Ghana. In addition to previous study^41^, we have also shown that NGS is a useful tool for the characterization of the virome in pediatric patients with CNS infection. Although it requires comprehensive laboratory conditions, a highly sensitive NGS-based pipeline has a great potential for analysis of clinical samples and to determine the possible viral implication in different pathological conditions.

## Methods

### Ethical issues

Informed consent was given by the parents of the study subjects prior to enrolment, and ethical approval was obtained from the Committee on Human Research, Publications and Ethics, School of Medical Science, Kwame Nkrumah University of Science and Technology, Kumasi, Ghana, and the Ethics Committee of the Ärztekammer Hamburg, Germany.

### Study setting and population

The present study is part of a larger malaria co-infections study in febrile pediatric inpatients in Ghana^8^. Briefly, the study took place in the pediatric ward of the Agogo Presbyterian Hospital (APH), located in the Asante Akim North District, Ghana. Agogo is a town of 36,797 inhabitants, located 80 km east of Kumasi. The Asante Akim North District area has a population of approximately 140.694 inhabitants (2010 Census Data, Ghana Statistical Service) with a tropical climate where malaria is highly endemic^37^. Children (n = 70) aged 1 month to 15 years with a tympanic temperature ≥38 °C and clinical suspicion for encephalitis/meningoencephalitis admitted to the pediatric ward were enrolled in the study. Healthy children (n = 120) from vaccination clinics from the study hospitals’ catchment area under 15 years of age with a tympanic temperature <37.5 °C and without clinical signs of infection were enrolled as a healthy cohort.

### Specimen collection

After a clinical examination, serum from febrile and afebrile study subjects has been collected. In addition, cerebrospinal fluid samples were obtained from inpatient children with fever and clinical suspicion of encephalitis/meningoencephalitis. Samples were collected before any medical treatment measures were initiated according to hospital guidelines (e.g. antimalarial or antibiotic drugs). In case of three patients included in this study we were able to collect serum samples 28 days after the initial antimalarial and/or antibiotics treatment. All methods were performed in accordance with the relevant guidelines and regulations of the institution.

### Novel human anelloviruses discovery in the cerebrospinal fluids and serum by next-generation sequencing

The patients’ cerebrospinal fluid and serum samples used for deep-sequencing were filtered through a 0.45-μm filter (Millipore, Darmstadt, Germany) to remove larger debris and some bacteria. 250 µl filtrates were treated with a mixture of nucleases (Turbo DNase, Ambion, Carlsbad, CA, USA; Baseline-ZERO, Epicenter, Madison, WI, USA; Benzonase, Novagen, San Diego, CA, USA; RNAse One, Promega, Fitchburg, WI, USA) to digest unprotected nucleic acids including host DNA/RNA. Enriched viral particles were then subjected to RNA/DNA extraction by using MagMAX™ Viral RNA Isolation Kit (Life Technologies, Carlsbad, California, USA) and QIAamp DNA Mini Kit (Qiagen, Hilden, Germany) according the manufacturer’s instructions. A negative water control which is an integrated part of our pipeline has been used for each run. Reverse transcription and cDNA synthesis were performed using 12 μl extracted RNA mixed with 100 pmol of random primer with a 20 bp fixed 5′ end sequence and at the 3′ a random nonamer (CCAGATGCCATCCAAGTGACNNNNNNNNN) and incubated at 72 °C, 2 min. First strand synthesis was done in a reaction mix consisting 1 µl SuperScript™ III reverse transcriptase, 1 µl dNTP (10 mM each; Qiagen), 5 × first-strand extension buffer and 10 mM dithiothreitol incubated at 25 °C for 10 min, followed by 50 °C incubation for 1 h and 70 °C for 15 minutes. The second strand reaction was carried out by incubation with 20 pmol of random primer, 2 µl 10x Klenow buffer and 5U Klenow Fragment (New England Biolabs, USA) at 37 °C for 1 h. The dsDNA obtained was PCR amplified using 5 µl sample, 20 pmol of the fixed portion of the random primer (CCAGATGCCATCCAAGTGAC), 5U HotStart Taq DNA Polymerase (Qiagen), 2.5 mM MgCl2, 0.2 mM dNTPs, and 1 X PCR buffer. Temperature cycling was performed according the manufacturer’s instructions except the annealing temperature (59°) and cycles (25). The random RT-PCR product DNA and extracted DNA were used for library preparation by using QIAseq FX DNA Library Kit (Qiagen, Germany) with double index barcode labeling according the manufacturer’s instructions. Library concentration was then measured using Qubit and Bioanalyzer instruments. Next generation sequencing was performed on a MiSeq Illumina platform using 2 × 300 bp paired-end sequencing. Resulting raw reads were first qualitatively checked, trimmed of adaptor sequences and filtered to remove polyclonal and low quality reads (<55 bases long) using CLC workbench (Qiagen). The remained reads per each pool were de-novo assembled separately using Trinity v2.6.6^42^ and Geneious v11 (Biomatters, New Zealand). The assembled contigs and unassembled singlets were compared with a non-redundant and viral proteome database using BLASTx and E-value cut-off 0.001. The virus-like contigs and singlets were further compared to all protein sequences in non-redundant protein databases using DIAMOND v0.9.6^43^ and a default e-value cutoff of 0.001. Each putative viral reads have been further used for partial or complete genome recovery using Geneious v11.

### Complete genome amplification of the novel anelloviruses

The complete genome sequences of the discovered anellovirus species have been obtained from the raw data generated in MiSeq. In addition, the untranslated region of the circular anellovirus genomes, including the GC-rich region have been reamplified or filled the gaps using specific primers based on genome sequences from the MiSeq using Sanger sequencing technology (all primers’ sequences used for UTR amplifications and overlapping PCR are available upon request). Amplification was performed using HotStar*Taq* Plus Master Mix Kit (Qiagen, Hilden, Germany). The PCR was performed in a 25 µl volume containing 3 µl of sample DNA, 10 µl of HotStar*Taq* Master Mix, 2 µl of each primer (20 pmol), and ddH_2_O up to 25 µl using a cycling condition as follows: denaturation at 95 °C for 15 min; 45 amplification cycles were performed at 94 °C for 20 s, 55–57 °C for 45 s, 72 °C for 1 min. Final extension was at 72 °C for 7 min.

### Prevalence of TTMV-G1–3 and TTV-G4 in cerebrospinal fluid and serum specimens

DNA from cerebrospinal fluid and serum of 70 pediatric patients with encephalitis/meningoencephalitis and serum of 120 afebrile healthy controls was extracted using a QIAamp DNA Mini kit (QIAGEN, Dusseldorf, Germany) according to the manufacturer’s instructions. TTMV-G1-3 and TTV-G4 species specific primers have been designed based on genome sequence obtained from the MiSeq analysis. PCR assays were performed with a final volume of 25 μl reaction mixture consisting of 3 μl extracted DNA, 10 µl HotStarTaq Master Mix, 1.5 µl of each primer (20 pmol), and ddH_2_O up to 25 µl using a cycling condition as follows: denaturation at 95 °C for 15 min; 45 amplification cycles were performed at 94 °C for 20 s, 55 °C for 30 s, 72 °C for 45 s. Final extension was at 72 °C for 10 min. Amplification products have been Sanger sequenced and compared with genomes obtained by MiSeq. The positive individuals have been further subjected to deep-sequencing and Sanger sequencing in order to obtain the complete genome.

### Genomic analysis

Genome sequence analysis, genomic organization and multiple alignments were performed using Geneious v11. Putative open reading frames were identified using ORF Finder (https://www.ncbi.nlm.nih.gov/orffinder/) and Geneious v11. Nucleotide and amino acid sequence identity and similarity were calculated using Sequence Demarcation Tool v1.2^44^, Geneious v11 and Sequence Editor, Database and Analysis Platform v1.3^45^.

### Phylogenetic relationship to other anelloviruses

Evolutionary relationships of TTMV-G1-3 and TTV-G4 with representative anelloviruses were determined by the construction of the phylogenetic trees based on nucleotide and amino acid sequences of the putative ORF1 gene. The initial ORF1 protein data set was pruned of divergent and ambiguously aligned blocks by using Gblocks program v0.91b^46^ with default parameters. The phylogenetic tree based on Gblock trimmed amino acid sequences and completes ORF1 nucleotide sequences were inferred using the Bayesian Markov chain Monte Carlo (MCMC) approach available in BEAST v1.8^47^ and maximum likelihood (ML) method implemented in PhyML^48^. Analyses were performed under the best fit nucleotide and amino acid substitution model identified as GTR + Г + I and LG + Г + I using Akaike information criterion as the model selection framework in jModelTest 2^49^. In order to assess the robustness of each node, a bootstrap resampling process was performed (1,000 replicates), again using the NNI branch-swapping method available in PhyML. All TTMV and TTV genomes from this study were confirmed as non-recombinant by using the various methods for recombination detection implemented in RDP4^50^.

### Nucleotide sequence accession number

Forty-two complete genome sequences of TTMV and TTV obtained in this study have been deposited in the GenBank database under accession numbers MH017546-MH017587.

## Supplementary information

Supplementary informations

## References

[1] World Health Organization, 2016. Global Health Observatory (GHO) data. Available at: http://www.who.int/gho/child_health/en/. (Accessed April 4, 2018).

[2] Liu L (2016). Global, regional, and national causes of under-5 mortality in 2000-15: an updated systematic analysis with implications for the Sustainable Development Goals. Lancet.

[3] Britton PN, Dale RC, Booy R, Jones CA (2015). Acute encephalitis in children: Progress and priorities from an Australasian perspective. J Paediatr Child Health.

[4] Mallewa M (2013). Viral CNS infections in children from a malaria-endemic area of Malawi: a prospective cohort study. Lancet Glob Health.

[5] Snow RW (1997). Relation between severe malaria morbidity in children and level of Plasmodium falciparum transmission in Africa. Lancet.

[6] Benjamin LA (2011). Human parvovirus 4 as potential cause of encephalitis in children, India. Emerg Infect Dis.

[7] Chan BK, Wilson T, Fischer KF, Kriesel JD (2014). Deep sequencing to identify the causes of viral encephalitis. PloS One.

[8] Hogan, B. *et al*. Malaria Coinfections in Febrile Pediatric Inpatients: A Hospital-Based Study From Ghana. *Clin Infect Dis* cix1120, 10.1093/cid/cix1120 (2018). 10.1093/cid/cix1120 PMC5982794 29408951

[9] Collins PL, Huang YT, Wertz GW (1984). Nucleotide sequence of the gene encoding the fusion (F) glycoprotein of human respiratory syncytial virus. Proc. Natl. Acad. Sci. USA.

[10] Hijikata M, Takahashi K, Mishiro S (1999). Complete circular DNA genome of a TT virus variant (isolate name SANBAN) and partial ORF2 sequences implicating a great degree of diversity beyond genotypes. Virology.

[11] Okamoto, H. *et al*. Species-specific TT viruses in humans and nonhuman primates and their phylogenetic relatedness. *Virology* **277**, 368–378, 10.1006/viro.2000.0588. 10.1006/viro.2000.0588 11080484

[12] Takahashi K, Iwasa Y, Hijikata M, Mishiro S (2000). Identification of a new human DNA virus (TTV-like mini virus, TLMV) intermediately related to TT virus and chicken anemia virus. Arch Virol.

[13] Hrazdilová, K. *et al*. New species of Torque Teno miniviruses infecting gorillas and chimpanzees. *Virology* **487**, 207–14, 10.1016/j.virol.2015.10.016. 10.1016/j.virol.2015.10.016 26547037

[14] Biagini, P. *et al*. Virus taxonomy. Ninth report of the International Committee on Taxonomy of Viruses (ed. King, A. M. Q., Adams, M. J., Carstens, E. B. & Lefkowitz E. J.) 331–341 (Elsevier 2011).

[15] O’Meara WP, Mangeni JN, Steketee R, Greenwood B (2010). Changes in the burden of malaria in sub-Saharan Africa. Lancet Infect Dis.

[16] Murray CJ (2012). Global malaria mortality between 1980 and 2010: a systematic analysis. Lancet.

[17] Black RE (2010). Global, regional, and national causes of child mortality in 2008: a systematic analysis. The Lancet.

[18] Kwofie TB (2012). Respiratory viruses in children hospitalized for acute lower respiratory tract infection in Ghana. Virol J.

[19] O’Brien KL (2009). Burden of disease caused by Streptococcus pneumoniae in children younger than 5 years: global estimates. Lancet.

[20] Nakawesi JS, Wobudeya E, Ndeezi G, Mworozi EA, Tum-wine JK (2010). Prevalence and factors associated with rotavirus infection among children admitted with acute diarrhea in Uganda. BMC Pediatr.

[21] Gessner BD, Shindo N, Briand S (2011). Seasonal influenza epidemiology in sub-Saharan Africa: a systematic review. Lancet Infect Dis.

[22] Okamoto HT (1998). Molecular cloning and characterization of a novel DNA virus (TTV) associated with post transfusion hepatitis of unknown etiology. Hepatol Res.

[23] Biagini P (2009). Classification of TTV and related viruses (anelloviruses). Curr Top Microbiol Immunol.

[24] Biagini P (2006). Distribution and genetic analysis of TTV and TTMV major phylogenetic groups in French blood donors. J Med Virol.

[25] Ninomiya M, Takahashi M, Nishizawa T, Shimosegawa T, Okamoto H (2008). Development of PCR assays with nested primers specific for differential detection of three human anelloviruses and early acquisition of dual or triple infection during infancy. J Clin Microbiol.

[26] de Villiers EM, Schmidt R, Delius H, zur Hausen H (2002). Heterogeneity of TT virus related sequences isolated from human tumour biopsy specimens. J Mol Med (Berl).

[27] Bando M (2001). Infection of TT virus in patients with idiopathic pulmonary fibrosis. Respir Med.

[28] Zhang Y (2016). A novel species of torque teno mini virus (TTMV) in gingival tissue from chronic periodontitis patients. Sci Rep..

[29] Ng TFF, Dill JA, Camus AC, Delwart E, Van Meir EG (2017). Two new species of betatorqueviruses identified in a human melanoma that metastasized to the brain. Oncotarget.

[30] Leppik L (2007). *In vivo* and *in vitro* intragenomic rearrangement of TT viruses. J Virol.

[31] Hijikata M (1999). Genotypes of TT virus (TTV) compared between liver disease patients and healthy individuals using a new PCR system capable of differentiating 1a and 1b types from others. Arch Virol.

[32] Tanaka Y (2001). Genomic and molecular evolutionary analysis of a newly identified infectious agent (SEN virus) and its relationship to the TT virus family. J Infect Dis.

[33] Peters MA, Jackson DC, Crabb BS, Browning GF (2002). Chicken anemia virus VP2 is a novel dual specificity protein phosphatase. J Biol Chem.

[34] Maggi F, Bendinelli M (2010). Human anelloviruses and the central nervous system. Rev Med Virol.

[35] Okamoto H (2001). 2001. Heterogeneous distribution of TT virus of distinct genotypes in multiple tissues from infected humans. Virology.

[36] Maggi F (2003). TT virus in the nasal secretions of children with acute respiratory. J Virol.

[37] Krefis AC (2011). Modeling the relationship between precipitation and malaria incidence in children from a holoendemic area in Ghana. Am J Trop Med Hyg.

[38] McElvania TeKippe E (2012). Increased prevalence of anellovirus in pediatric patients with fever. PLoS One.

[39] De Vlaminck I (2013). Temporal response of the human virome to immunosuppression and antiviral therapy. Cell.

[40] Christensen JK (2000). Prevalence and prognostic significance of infection with TT virus in patients infected with human immunodeficiency virus. J Infect Dis.

[41] Kawada J (2016). Identification of Viruses in Cases of Pediatric Acute Encephalitis and Encephalopathy Using Next-Generation Sequencing. Sci Rep.

[42] Grabherr MG (2011). Full-length transcriptome assembly from RNA-seq data without a reference genome. Nat Biotechnol.

[43] Buchfink B, Xie C, Huson DH (2015). Fast and Sensitive Protein Alignment using DIAMOND. Nature Methods.

[44] Muhire BM, Varsani A, Martin DP (2014). SDT: a virus classification tool based on pairwise sequence alignment and identity calculation. PLoS One.

[45] Simmonds P (2012). SSE: a nucleotide and amino acid sequence analysis platform. BMC Res Notes..

[46] Talavera G, Castresana J (2007). Improvement of phylogenies after removing divergent and ambiguously aligned blocks from protein sequence alignments. Syst Biol.

[47] Drummond AJ, Suchard MA, Xie D, Rambaut A (2012). Bayesian phylogenetics with BEAUti and the BEAST 1.7. Mol Biol Evol.

[48] Guindon S (2010). New algorithms and methods to estimate maximum-likelihood phylogenies: assessing the performance of PhyML 3.0. Syst Biol.

[49] Darriba D, Taboada GL, Doallo R, Posada D (2012). iModelTest 2: more models, new heuristics and parallel computing. Nat Methods.

[50] Martin D, Rybicki E (2000). RDP: detection of recombination amongst aligned sequences. Bioinformatics.

