# Supplementary material for: Viral metagenomics revealed novel betatorquevirus species in pediatric inpatients with encephalitis/meningoencephalitis from Ghana
Source: Sci Rep. 2019 Feb 20;9:2360. doi: 10.1038/s41598-019-38975-z (PMC6382885; doi:10.1038/s41598-019-38975-z)
Supplement: Supplementary file 1 — Supplementary informations [file 41598_2019_38975_MOESM1_ESM.pdf]

Supplementary information

Viral metagenomics revealed novel betatorquevirus species in pediatric inpatients with encephalitis/meningoencephalitis from Ghana

Daniel Eibach[1,2,†], Benedikt Hogan[1,2,†], Nimako Sarpong[3], Doris Winter[1], Nicole S. Struck[1], Yaw Adu-Sarkodie[4], Ellis Owusu-Dabo[3], Jonas Schmidt-Chanasit[5,2,#], Jürgen May[1,2,#], Daniel Cadar[5,2,#,*]

[1]Department of Infectious Disease Epidemiology, Bernhard Nocht Institute for Tropical Medicine, Hamburg, 20359, Germany
[2]German Center for Infection Research, Hamburg-Borstel-Lübeck-Riems, 20359, Germany
[3]Kumasi Centre for Collaborative Research in Tropical Medicine, Kumasi, 40080, Ghana
[4]Department of Clinical Microbiology, Kwame Nkrumah University of Science and Technology, Kumasi, 40080, Ghana
[5]Department of Arbovirology, Bernhard Nocht Institute for Tropical Medicine, Hamburg, 20359, Germany

Supplementary Table 1. TTMV-G1-3 and TTV-G4 species specific PCR results in healthy control.

Sample ID	Age (in month)	Gender	TTMV-G1	TTMV-G2	TTMV-G3	TTV-G4
80268	23	female	-	-	-	-
80102	36	Male	-	-	-	-
80597	36	female	-	-	-	-
80132	23	male	-	-	-	-
80229	17	male	-	-	-	-
80127	47	male	-	-	-	-
80373	3	male	-	-	-	-
80529	11	male	-	-	-	-
80590	131	male	-	-	-	-
80474	2	female	-	-	-	-
80548	47	male	-	-	-	-
80540	108	male	-	-	-	-
80394	6	female	-	-	-	-
80467	108	male	-	-	-	-
80260	21	female	-	-	-	-
80509	13	male	-	-	-	-
80005	0	male	-	-	-	-
80441	19	male	-	-	-	-
80138	84	male	-	-	-	-
80121	22	female	-	-	-	-
80370	18	male	-	-	-	-
80526	23	male	-	-	-	-
80332	23	male	-	-	-	-
80576	47	male	-	-	-	-
80206	47	male	-	-	-	-
80057	13	female	-	-	-	-
80032	15	female	-	-	-	-
80398	60	male	-	-	-	-
80342	11	female	-	-	-	-
80198	23	female	-	-	-	-
80028	23	male	-	-	-	-
80008	9	female	-	-	-	-
80199	9	female	-	-	-	-
80479	3	male	-	-	-	-
80414	60	female	-	-	-	-
80256	15	male	-	-	-	-
80106	47	female	-	-	-	-
80126	108	female	-	-	-	-
80233	13	male	-	-	-	-
80202	11	female	-	-	-	-
80515	155	female	-	-	-	+

80412	47	male	-	-	-	-
80416	23	female	-	-	-	-
80574	144	male	-	-	-	-
80511	23	male	-	-	-	-
80171	23	female	-	-	-	-
80481	36	male	-	-	-	-
80577	84	male	-	-	-	-
80586	23	female	-	-	-	+
80301	23	female	-	-	-	-
80099	5	female	-	-	-	-
80218	36	female	-	-	-	-
80425	168	male	-	-	-	-
80419	3	male	-	-	-	-
80019	36	female	-	-	-	+
80109	84	female	-	-	-	-
80305	11	male	-	-	-	-
80184	72	female	-	-	-	-
80572	180	male	-	-	-	-
80203	47	female	-	-	-	-
80534	18	female	-	-	-	-
80267	17	male	-	-	-	-
80037	18	female	-	-	-	-
80194	18	female	-	-	-	-
80004	47	male	-	-	-	-
80090	19	male	-	-	-	-
80545	3	male	-	-	-	-
80292	19	female	-	-	-	-
80593	36	female	-	-	-	-
80389	2	female	-	-	-	-
80491	11	female	-	-	-	-
80382	2	female	-	-	-	-
80363	13	female	-	-	-	-
80456	15	female	-	-	-	-
80042	3	female	-	-	-	-
80252	15	female	-	-	-	-
80073	23	female	-	-	-	-
80224	7	male	-	-	-	-
80265	7	male	-	-	-	-
80478	1	female	-	-	-	-
80177	18	female	-	-	-	-
80217	23	female	-	-	-	-
80310	36	female	-	-	-	-
80261	17	male	-	-	-	-
80523	9	female	-	-	-	-
80333	7	female	-	-	-	-
80354	23	male	-	-	-	-
80088	19	female	-	-	-	-

80244	72	female	-	-	-	-
80313	95	female	-	-	-	-
80471	72	male	-	-	-	-
80516	36	female	-	-	-	-
80013	6	male	-	-	-	-
80533	3	female	-	-	-	-
80457	3	male	-	-	-	-
80517	23	female	-	-	-	-
80055	13	female	-	-	-	-
80445	47	female	-	-	-	-
80077	23	female	-	-	-	-
80134	60	male	-	-	-	-
80375	2	male	-	-	-	-
80111	23	male	-	-	-	-
80361	23	female	-	-	-	-
80251	155	male	-	-	-	-
80386	7	female	-	-	-	-
80246	95	male	-	-	-	-
80145	6	male	-	-	-	-
80431	23	female	-	-	-	-
80550	60	female	-	-	-	-
80483	5	male	-	-	-	-
80315	2	female	-	-	-	-
80205	36	male	-	-	-	-
80307	47	female	-	-	-	-
80041	19	male	-	-	-	-
80480	84	female	-	-	-	-
80269	23	female	-	-	-	-
80427	72	female	-	-	-	-
80047	7	male	-	-	-	-
80002	15	female	-	-	-	-
80512	5	female	-	-	-	-

Supplementary Table 2. Parasitic and/or bacterial infections detected in TTMV-G1-3 and TTV-G4 negative hospitalized febrile pediatric patients with CNS infections.

Patient ID	Age (in months)	Gender	*P. falciparum* parasitemia /µl	Blood culture isolate	CSF culture isolate
700606	55	female	179	-	-
700625	28	female	108114	-	-
700636	23	female	-	-	-
700643	24	male	338324	-	-
700657	29	female	139950	-	-
700658	18	female	332304	-	-
700670	76	male	-	-	-
700696	27	male	14830	-	-
700703	20	female	-	-	-
700705	25	male	237293	-	-
700739	7	male	2408	-	-
700766	16	female	202410	-	-
700771	7	male	32432	-	-
700773	18	male	228672	-	-
700790	30	female	-	-	-
700809	47	male	4118	-	-
700816	32	male	177912	-	-
700836	38	male	132055	-	-
700837	26	female	5232	-	-
700872	4	female	-	-	-
700930	36	female	-	-	-
700931	36	male	25433	-	-
700959	37	female	50267	-	-
700981	63	female	105948	-	-
700985	26	female	75484	-	-
701004	30	male	78631	-	-
701027	27	male	403497	-	-
701032	24	male	719264	-	-
701040	29	male	67656	-	*Klebsiella oxytoca*
701049	18	female	-	-	-
701050	19	female	33210	-	-
701054	24	female	92652	-	-
701059	25	female	123876	-	-
701063	47	male	-	-	-
701070	8	female	49516	-	-
701076	22	male	34956	-	-
701077	28	male	-	-	-
701082	8	female	-	-	-
701113	39	male	166440	-	-
701133	19	female	46569	-	-

701135	50	female	36354	-	-
701162	27	male	89310	-	-
701181	30	female	432224	-	-
701222	60	male	104340	-	-
701225	72	male	403	-	-
701232	8	female	104064	-	-
701233	23	female	-	-	-
701240	80	female	159840	-	-
701310	10	female	28980	-	-
701325	36	female	-	-	-
701345	48	male	232128	-	-
701368	24	male	465300	-	-
701414	49	male	17208	-	-
701538	14	male	-	-	-
701553	44	female	305890	-	-
701622	29	male	2948	-	-
